# Editorial: Intermediate filaments structure, function, and clinical significance

**DOI:** 10.3389/fcell.2022.1103110

**Published:** 2022-11-30

**Authors:** Dolores Pérez-Sala, Ming Guo

**Affiliations:** ^1^ Department of Structural and Chemical Biology, Centro de Investigaciones Biológicas Margarita Salas, CSIC, Madrid, Spain; ^2^ Department of Mechanical Engineering, Physics of Living Systems Center, Center for Multi-Cellular Engineered Living Systems, Massachusetts Institute of Technology, Cambridge, MA, United States

**Keywords:** Vimentin, desmin, lamin, mechanosensing, cytoskeletal interplay, intermediate filaments in pathophysiology, epithelial–mesenchymal transition (EMT), extracellular vimentin

The cytoskeleton is a highly complex network formed by three main types of structures, namely, microtubules, actin microfilaments and intermediate filaments (IF). Cytoskeletal structures can be viewed as macromolecular machines implementing cell dynamics, making possible a plethora of processes including intracellular transport, contractility, migration and division. In this complex and dynamic mesh, IF are not merely structural scaffolds giving support to cellular organelles. Instead, they function as integrators of cytoskeletal networks and link mechanical and chemical cues to signal transduction, regulation of cell morphology and proliferation, organelle homeostasis and gene transcription, among other essential cellular processes (Etienne-Manneville, 2018; Vahabikashi et al., 2022).

In contrast to actin and tubulin, coded by a low number of genes, IF proteins are the products of over 60 different genes, and their versatility provides structures specific for certain subcellular compartments, as in the case of nuclear lamins, or cell-type-specific flavors, with proteins “tailored” for expression in mesenchymal cells (vimentin), muscle cells (desmin), neurons (neurofilaments) or astrocytes (glial fibrillary acidic protein) (Eriksson et al., 2009).

As IF mediate critical cellular functions, their disruption by mutation or environmental factors leads to disease, as in aging syndromes (lamins), cardiac and skeletal muscle myopathies (desmin), skin diseases (keratins), neurodegenerative disease (GFAP), or cancer progression, fibrosis and infection (vimentin) (Eriksson et al., 2009).

Articles collected in the *Intermediate filaments structure, function, and clinical significance* Topic include cutting-edge original research reports, state-of-the-art review articles covering the essentials of the field, and trends and perspectives towards the key open questions in IF biology and biophysics.

As IFs are key integrators of cellular structures, several articles deal with their crosstalk with other cytoskeletal components. Cellular mechanoresponsiveness, the process by which cells sense and adapt to mechanical changes, was traditionally considered an actin-orchestrated process. Nevertheless, it is becoming clear that mechanosensitivity requires the close interplay between the three cytoskeletal filament systems, with IF playing a central role (Ndiaye et al.) Mechanical stability is particularly important in epithelia. The article by Moch et al., illustrates the crosstalk between adherens junction-associated actomyosin cytoskeleton and the desmosome-anchored keratin IFs in regulating epithelial mechanics. They show how cortical tension affects the complex desmosome structure, modulating desmosome formation and protein composition, thus evidencing a novel desmosomal mechanoresponsive pathway which senses alterations in force balance.

Alterations in cell-cell adhesion and cell mechanics are key during epithelial-mesenchymal transition (EMT), the process by which cancerous epithelial cells acquire tumorigenic potential with increased migratory and invasive capacities leading to metastasis. The type III IF protein vimentin is a marker and player of this process (Battaglia et al., 2018; Ridge et al., 2022), but the molecular mechanisms involved are largely unknown. Sivagurunathan et al., show that vimentin induction in an epithelial cell line alters keratin networks and disrupts desmosomes, reducing intercellular forces and stiffness. At the same time, vimentin activates transcription of genes involved in cell locomotion, thus creating the perfect scenario for increased cell migration and therefore, tumorigenic potential. Importantly, vimentin also influences cell migration by controlling extracellular matrix remodeling and interactions. The review by Ostrowska-Podhorodecka et al., provides an expert view of the multiple roles of vimentin in this process. Vimentin controls collagen deposition and extracellular matrix structure. Moreover, it interacts with adhesion receptors such as integrins, regulating the growth, maturation and strength of integrin-dependent adhesions, thus affecting cell migration through connective tissues. Importantly, the work by Hakibilen et al., identifies a novel role for desmin, a muscle-specific type III IF protein highly homologous to vimentin, in cell-matrix adhesion, which is critical for strength transmission, satellite cell migration and muscle regeneration. In this case, desmin appears to promote cell adhesion and limit migration. This work suggests a potential involvement of adhesion and migration defects in the pathogenesis of desminopathies, which could lead to novel therapeutic approaches. Therefore, the role of vimentin and other type III IFs in cell adhesion and migration in physiology and pathophysiology can be highly complex and potentially dependent on the cell type and the host tissue. In two models of intestinal inflammation and tumorigenesis (Wang et al.), vimentin appears to play a protective role, since vimentin-null (*Vim−/−*) mice challenged with dextran sodium sulfate display higher generation of reactive oxygen and nitrogen species and worse colitis than the wild type mice, and develop more and larger tumors in the tumorigenesis model. Conversely, vimentin plays a key role in lung diseases, not only during EMT occurring in lung cancer, but in infectious diseases and in lung fibrosis (Ridge et al., 2022). The review by Surolia and Antony provides a deep analysis of the roles of vimentin in lung pathophysiology, covering processes from lung injury and acute respiratory syndrome, to respiratory infections, both viral and bacterial, as well as chronic lung diseases, such as fibrosis and chronic obstructive pulmonary disease. This clinically-oriented review also examines the therapeutic strategies targeting vimentin, including anti-vimentin compounds and antibodies, that could be relevant for lung disease.

The multifaceted roles of vimentin are supported by its great versatility. Vimentin is a hub for multiple posttranslational modifications that finely tune its assembly mode, cellular localization and interactions (Viedma-Poyatos et al., 2020; Griesser et al., 2021). Therefore, the diverse vimentin proteoforms can perform amazingly different functions. The structure of vimentin is not fully understood, and multiple approaches have been employed to characterize it, both *in vitro* and in cells. Most advances have improved our comprehension of the central alpha-helical domain (rod) and the N-terminal domain (head) of the protein (Eldirany et al., 2021). However, the disposition of the intrinsically disordered C-terminal domain (tail), involved in cytoskeletal crosstalk, remains unsolved. Based on epitope accessibility analyses, Lois-Bermejo et al., propose that at least two different conformations of vimentin tail coexist in cells, with a “packed” conformation predominant in perinuclear filaments and stress-induced bundles, and a “loose or extended” conformation mainly present in peripheral filaments, which could arise by tail phosphorylation, and potentially influence vimentin assembly and interactions. Besides its presence in long cytoplasmic filaments, vimentin can exist in small particles at the migration front of cells, and even appear at the cell surface or in the extracellular medium in various non-filamentous forms. Indeed, extracellular vimentin can act as a ligand for various agents, including pathogens, and vimentin present in exosomes influences cell migration (Ramos et al., 2020). Thalla et al., observe extracellular vimentin on the “back” of activated macrophage-like cells as small fragments forming agglomerates on the cell surface. This polarized release of vimentin appears to enhance migration and phagocytic activity of these cells, raising new exciting avenues for the study of the involvement of vimentin in immune cells functions.

Nuclear IF, the lamins, are key for nuclear integrity and gene transcription, and mutations in the corresponding genes cause the diseases known as laminopathies, characterized by severe clinical manifestations, either organ-selective or systemic, as in the premature aging syndromes (Worman and Bonne, 2007). Lamins line the inner nuclear membrane and play key roles in integration of signals and transmission of forces between the cytoplasm and the nucleus. Shaw et al., investigate how disease-causing *LMNA* mutations alter nucleo-cytoskeletal coupling in a *Drosophila* model of muscular dystrophy. They identify lamin residues important for connecting the nucleus to the cytoskeleton, unveiling that specific mutations cause different defects in subcellular mechanics, with potentially distinct pathogenic implications. In addition, the mutant proteins themselves can exert deleterious effects in cells, as a consequence, among other factors, of impaired protein degradation. Indeed, West et al., show that a mutation in the *LMNA* gene causes the mutant proteins to overload and impair the ubiquitin proteasome system. Under these conditions, compensatory enhanced autophagy or restoration of protein degradation, achieved by a chemical chaperone, help maintaining the levels of mutant proteins, and therefore cell homeostasis. Consequently, the authors propose the protein degradation machinery as a therapeutic target in laminopathies associated with proteostasis defects.

In summary, the articles contained in this Topic highlight the complex biological and pathophysiological roles of IF, reinforcing their importance as central players in cell and tissue homeostasis, as well as their promise as therapeutic targets.
